# Supplementary material for: Modeling and simulation of neocortical micro- and mesocircuitry (Part I, anatomy)
Source: eLife. 2026 Jan 20;13:RP99688. doi: 10.7554/eLife.99688 (PMC12818870; doi:10.7554/eLife.99688)
Supplement: Supplementary file 1. [file elife-99688-supp1.docx]

**Descriptions of individual validations and their results**

We began by validating that the cortical layers placed in the atlas formed a stack of continuous volumes. To that end, we calculated the fraction of voxels in each layer that were directly adjacent to another layer. As expected, adjacent voxels were found only between adjacent layers. Fractions of adjacent voxels decreased in lower layers as they are generally thicker and due to the curved geometry of the volume. Additionally, we tested that layers are spatially continuous by confirming that for each voxel there was at least one neighboring voxel in the same layer. This was the case for over 99.99% of the voxels with the handful of violating voxels limited to the periphery of the modeled volume (not shown).

To validate the neuronal composition of the model, we then compared the densities of excitatory and inhibitory neurons placed in the model against the input constraints. The vertical density profiles matched the input robustly, with minor numerical differences resulting from the need to round the number of neurons to place in a voxel to the nearest integer.

The biologically correct placement of neuronal morphologies was first validated by visual inspection of renderings of neurons in the context of the atlas (Figure 2 - supplement 5). More quantitatively, we considered for each neuron its placement score that quantifies to what degree manually identified morphological features reach the correct layers and neurites remain within the model volume (see Figure 2 - supplement 4; Methods). Specifically, we ensured that the fraction of neurons with poor placement score remained below 1%.

Connectivity was validated in terms of the following aspects: We ensured that the mean bouton density on axons matched biological reference data (Figure 4 - supplement 3A). A mismatch was only observed for Chandelier Cells in layers 2/3 and 4. This is a consequence of the highly specific connectivity of those m-types targeting only axon initial segments of postsynaptic neurons, which is challenging to model based on axonal appositions. Further, we ensured that the mean number of synapses per connection in m-type-specific pathways matches the biological reference (Figure 4 - supplement 3B). For mid-range connectivity, we ensured that the topographical mapping between subregions and laminar profiles of synapse locations matches the specified references (Figure 4 - supplement 3D, E). For the union of local and mid-range connectivity, we validated the overall density of synaptic connections between subregions (Figure 4 - supplement 3C). Finally, for thalamic inputs we validated their overall strengths and layer specificities by comparing the laminar profiles of their synapse locations to reference data (Figure 6 - supplement 2).
