# Supplementary material for: Modeling and simulation of neocortical micro- and mesocircuitry (Part I, anatomy)
Source: eLife. 2026 Jan 20;13:RP99688. doi: 10.7554/eLife.99688 (PMC12818870; doi:10.7554/eLife.99688)
Supplement: Supplementary file 2. [file elife-99688-supp2.docx]

**Inherited assumptions**

We assume that the published data sources and modelling steps (Table 1) are correct and consequently inherit their assumptions. Below, we will only list the assumptions made on top of that.

**Data assumptions**

We make a number assumptions related to generalizing input data

- We assume the juvenile rat nbS1 volume is a uniformly scaled-down version of the adult nbS1.
- We assume no significant variability between nbS1 regions in terms of: Cell density layer profiles, relative layer thickness, neuronal morphologies, bouton densities, number of synapses per connection in pathways.
- We assume the relative strengths of connectivity and structure axonal targeting is comparable in nbS1 regions between mouse and rat.
- We assume that the VPM to barrel cortex projection in adult rat is representative for a general core-type, feedforward thalamic input in juvenile rat. Similarly, POm to barrel cortex for matrix-type, feedback input.
- We assume mouse thalamo-cortical projection axons are informative for the structure of the rat thalamo-cortical projection system.

**Structuring assumptions**

- We assume that grouping of cortical neurons into 60 morphological classes is also useful for the description of the neuronal composition (cell density profiles) and the synaptic connectivity between them.
- We assume that the eight established subregions provide a parcellation that is useful for the description of mid-range connectivity in the model.

**Modeling assumption**

- We assume that purely vertical cell density profiles capture all relevant details of neuronal composition.
- We assume that the union of synaptic connections from two separate algorithms accurately describes the connectivity at all scales that are relevant in the model. Specifically, we assume there is no "midrange gap" remaining between the two algorithms.
- We assume that thalamo-cortical axons do not target specific classes of neurons, beyond what is given by their layer profiles.
- We assume the horizontal spread of thalamo-cortical axons can be captured by a Gaussian profile for the synapses formed.
