# Supplementary material for: Modeling and simulation of neocortical micro- and mesocircuitry (Part I, anatomy)
Source: eLife. 2026 Jan 20;13:RP99688. doi: 10.7554/eLife.99688 (PMC12818870; doi:10.7554/eLife.99688)
Supplement: Supplementary file 4. [file elife-99688-supp3.docx]

| (1) |  |
| --- | --- |
| Prediction | The complexity of connectivity observed in EM reconstructions arises from  neuronal morphologies and their 3D placement in ways that are not captured  by simpler connectivity rules. |
| Importance | Complex higher-order network features observed in electron microscopy reconstructions emerge in our model. We predict that they cannot emerge from simple wiring rules (used for point neuron models for example) based on input and output degrees, connection probability between different m-types or the distance between neurons, even when individual pathways formed between morphological types are taken into account.  This brings us one step closer to finding the “wiring rules” of the brain, which implement a network that is robust, flexible and efficient.  This has impact beyond the study of the cortex itself addressing a core question in machine learning of what constitutes a sparse, efficient and robust network for learning. |
| Novelty | Our methods reveal these results by taking higher order interactions into account. Indeed, this results are invisible to analyses that take into account only pairwise interactions (e.g., node degree).  Furthermore, to our knowledge, currently there is no basic mathematical algorithm to build networks of this type (Young et al. (2017); Unger and Krebs (2024)). |
| Experiment | This finding has been now verified in the MICrONS dataset (MICrONS-Consortium  et al., 2021) as well as in other species in Egas Santander et al. (2024). |

| (2) |  |
| --- | --- |
| Prediction | The importance of a neuron within a network changes across scales and is co-determined by its participation in higher-order motifs. |
| Importance | It has long been known that neurons in different cortical layers form different fractions of local and mid-range connections. We developed quantitative metrics that capture the centrality of a neuron at both scales, i.e. its importance for information processing within a local circuit and distributed across regions. We confirmed that it differs between layers, but also found that it is affected by qualitative factors. That is, not only the number of connections it participates in matters, but also the way they are organized topologically. This is a step towards closing the gap between our theories of cortical computation at the microcircuit and at the individual neuron level. |
| Novelty | This is a new view of the computational role of individual cortical layers that goes beyond the established theories of input- and output layers. The qualitative aspects of connectivity considered lead to the question of *efficiency* of connectivity at the topological level, i.e. how a given function can be implemented with the lowest number of synaptic connections. This is further explored in Egas Santander et al., 2024 (iScience). Finally, we highlighted a structural differences between mid-range connectivity (highly heterogeneous) versus local connectivity (mostly homogeneous). |
| Experiment | This diversity of centrality across dimensions has been verified in the MICrONS dataset  (MICrONS-Consortium et al., 2021) in Reimann et al. (2024) and could continue to be  tested in future detailed reconstructions. |

| (3) |  |
| --- | --- |
| Prediction | Interactions between spatially separated microcircuits is mediated by pairs of neurons in layer 5 |
| Importance | It has been demonstrated before that cortical circuits receive more mid-range cortico-cortical than local connections. Yet, the number of non-local cortical neurons is vastly higher than the number of local neurons. This leads to a question that can only be answered with quantitative information at large spatial scales: To what degree can pairs of neurons in different regions or microcircuits interact closely enough to form units of information processing, such as distributed assemblies? Or is single neuron functionality restricted to the local circuit level? In other words, is the cortical network a hierarchical network of local networks or a single, highly distributed one? We showed that the vast majority of pairwise interactions are mediated via stereotypical motifs involving layer 5 neurons, favoring the hierarchical view. |
| Novelty | In a network of this scale, the study of all paths between pairs of nodes is infeasible.  The perspective of higher order interactions gave us a notion of a core of a network, that allowed us to restrict our analysis to a subset of central-nodes in this high dimensional sense, making a path distance based study possible.  Moreover, we found a strong directionality in this “core” implemented differently for the local and mid-range connectivity, indicating different functionality. |
| Experiment | This result could be tested experimentally once EM reconstructions are obtained for a volume 25 time larger than the one measure in the MICrONS data set. |

| (4) |  |
| --- | --- |
| Prediction | We predict the quantitative and spatially specific targeting rules of different neuron types, finding them to be very different between types. |
| Importance | The insights outlined above describe a network of high complexity and non-random structure; calling for the study of the mechanisms giving rise to it. These mechanisms range from general and large-scale to specific and micro-scale. Our work allowed us to predict the relative importance of the different mechanisms shaping connectivity (see Discussion in the original manuscript). This allows us to improve our intuitive understanding of the roles of different types of neurons in cortical processing. |
| Novelty | Such dissociation between the different possible mechanisms can really only be achieved by having in unison electron-microscopic analysis and a flexible and modifiable large scale model in which to recreate this.  Our work provides exactly the platform to do this. Previously people described specificity only by the resulting connectivity. In contrast, we were able to make inferences about the mechanisms which give rise to the connectivity. Specifically, we found that even apparently very specific connectivity can arise from the shapes of neurons only. |
| Experiment | This prediction is on an abstract level. It could be confirmed or rejected by linking connectivity targeting of various neuron types to more specific molecular and gene expression factors, then investigating the spatial scales at which the mechanisms operate. |

| (5) |  |
| --- | --- |
| Prediction | The complexity of connectivity observed in EM reconstructions needs an atlas supporting cortical anatomical variability. |
| Importance | Difference in layer height and the curvature of the atlas volume at a given point in the cortex changes the cell composition and the density of connections in that region.  This may have either functional consequences or morphological consequences if each layer has a specific computational purpose to implement a robust functioning circuit across anatomical differences. |
| Novelty | Our atlas-based model allowed us to use geometry metrics quantifying the curvature and depth of different locations of the cortex. This allowed us to provide, to our knowledge, the first quantitative predictions of the effect of cortical anatomical variability in neuronal density and network density. |
| Experiment | Electron microscopic connectome initiatives, such as MICrONS-Consortium et al., 2021 can be targeted at regions with different curvature, followed by comparison of the results. |
